# *De novo* transcriptome assembly of the wild relative of tea tree (*Camellia taliensis*) and comparative analysis with tea transcriptome identified putative genes associated with tea quality and stress response

**DOI:** 10.1186/s12864-015-1494-4

**Published:** 2015-04-15

**Authors:** Hai-Bin Zhang, En-Hua Xia, Hui Huang, Jian-Jun Jiang, Ben-Ying Liu, Li-Zhi Gao

**Affiliations:** Plant Germplasm and Genomics Center, Germplasm Bank of Wild Species in Southwest China, Kunming Institute of Botany, the Chinese Academy of Sciences, Kunming, 650204 China; University of Chinese Academy of Sciences, Beijing, 100039 China; Tea Research Institute, Yunnan Academy of Agricultural Sciences, Menghai, 666201 China

## Abstract

**Background:**

*Camellia taliensis* is one of the most important wild relatives of cultivated tea tree, *C. sinensis*. The species extensively occupies mountainous habitats representing a wide-range abiotic tolerance and biotic resistance and thus harbors valuable gene resources that may greatly benefit genetic improvement of cultivated tea tree. However, owning to a large genome size of ~3 Gb and structurally complex genome, there are fairly limited genetic information and particularly few genomic resources publicly available for this species. To better understand the key pathways determining tea flavor and enhance tea tree breeding programs, we performed a high-throughput transcriptome sequencing for *C. taliensis*.

**Results:**

In this study, approximate 241.5 million high-quality paired-end reads, accounting for ~24 Gb of sequence data, were generated from tender shoots, young leaves, flower buds and flowers using Illumina HiSeq 2000 platform. *De novo* assembly with further processing and filtering yielded a set of 67,923 transcripts with an average length of 685 bp and an N50 of 995 bp. Based on sequence similarity searches against public databases, a total of 39,475 transcripts were annotated with gene descriptions, conserved protein domains or gene ontology (GO) terms. Candidate genes for major metabolic pathways involved in tea quality were identified and experimentally validated using RT-qPCR. Further gene expression profiles showed that they are differentially regulated at different developmental stages. To gain insights into the evolution of these genes, we aligned them to the previously cloned orthologous genes in *C. sinensis*, and found that considerable nucleotide variation within several genes involved in important secondary metabolic biosynthesis pathways, of which flavone synthase II gene (*FNSII*) is the most variable between these two species. Moreover, comparative analyses revealed that *C. taliensis* shows a remarkable expansion of LEA genes, compared to *C. sinensis*, which might contribute to the observed stronger stress resistance of *C. taliensis*.

**Conclusion:**

We reported the first large-coverage transcriptome datasets for *C. taliensis* using the next-generation sequencing technology. Such comprehensive EST datasets provide an unprecedented opportunity for identifying genes involved in several major metabolic pathways and will accelerate functional genomic studies and genetic improvement efforts of tea trees in the future.

**Electronic supplementary material:**

The online version of this article (doi:10.1186/s12864-015-1494-4) contains supplementary material, which is available to authorized users.

## Background

The genus *Camellia* is composed of over 110 taxa [1], of which *C. sinensis* (L.) O. Kuntze is often commercially used as a source of the beverage tea. *C. taliensis,* commonly described as “wild” tea plant by the local people of its growing areas, is one of the most important wild relatives of the cultivated tea, and they together belong to the section *Thea*. They both are monoecious, insect-pollinated, and outcrossing species, but differ from each other primarily in the number of locules and the sizes of flowers and leaves. The number of locules per ovary is five in *C. taliensis*, while three for *C. sinensis*. The *C. sinensis* has high cross-compatibility with most of its allied species in the genus *Camellia*, especially with the *C. taliensis* [2]. Because of its fascinated aftertaste and close relationship with the cultivated tea, *C. taliensis* has also been consumed instead of common tea by local people in some regions of Asia, particularly in Yunnan Province of China [1]. *C. taliensis* usually grows in the mountainous evergreen broad-leaved forests at altitudes from 1,300 to 2,400 m, and is mainly distributed in southwestern Yunnan of China as well as adjacent regions including northern Myanmar and Thailand. Notably, the *C. taliensis* population was also found on ‘Mengku’ Snow Mountain, Yunnan, China, at the altitude of 2,750 m, implying an extremely strong stress resistance in *C. taliensis* [3]. Thus, it may harbor abundant gene resources that have great potential to enhance genetic improvement of cultivated tea in the future. The transcriptome of *C. sinensis* has been sequenced [4,5], and many tea quality or cold tolerance-related genes have been identified in this species. However, limited genomic resources are available for *C. taliensis*, probably due to its large genome size of around 3 Gb [6]. Until now, there are only 390 protein sequences, 64 nucleotide sequences and 137 gene sequences of *C. taliensis* in GenBank database (as of August 2014). They are apparently insufficient for mining functional genes encoding enzymes associated with important secondary metabolic pathways within *C. taliensis* and the genetically improving the flavor and yield of cultivated tea tree.

Transcriptome sequencing using next-generation sequencing technologies is a fast and cost-effective approach to generate genome-scale sequence resources [7,8], and thus has increasingly been employed in more and more plants. Particularly for those lacking a sequenced reference genome or non-model species, it provides an efficient and prior way to investigate patterns of gene expression, discover novel genes, and obtain a large number of genetic markers. Currently, three next-generation sequencing (NGS) platforms, including Roche 454, Illumina Genome Analyzer and Life Technologies SOLiD, are available and they can generate massive sequence reads at an extraordinary depth. In terms of the sensitivity, accuracy and throughput, these platforms have different advantages and limitations. Among them, Illumina sequencing technology, which generates large-scale reads (75–150 bp) with a high sequencing coverage at lower costs, has extensively been used for *de novo* transcriptome studies [9-11].

In this study, we present the first transcriptome of *C. taliensis* using the next-generation sequencing platform Illumina. The large EST dataset will remarkably enlarge genomic resources of *C. taliensis* available in the public database. The obtained data have led to the identification of a number of candidate genes and functional elements determining major metabolic pathways associated with tea-quality and stress resistance that are of great importance to enhance efforts for genetic improvement of the cultivated tea tree.

## Results and discussion

### Sequencing and *de novo* assembly of the *C. taliensis* transcriptome

**Figure 1:**
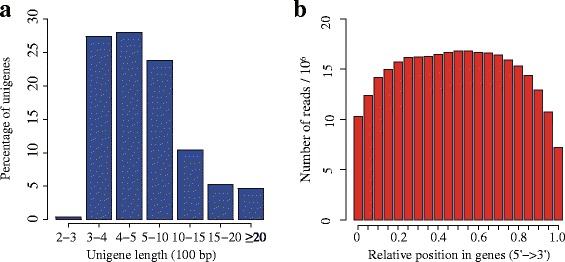
Summary of the *C. taliensis* transcriptome assembly. **(a)** Size distribution of the assembled unigenes. **(b)** Random distribution of the sequencing reads in the unigenes. The x-axis indicates the relative position in the unigenes. The orientation is from 5’ end to 3’ end.

To comprehensively construct the complete transcriptome of *C. taliensis*, four tissues representing various development stages, including tender shoots, young leaves, flower buds and flowers, were harvested for RNA isolation. Following the Illumina manufacturer’s instructions (Illumina, San Diego, CA, USA), the shotgun libraries were constructed and used for sequencing with the Illumina High-Seq 2000 platform. In total, ~241.5 million paired-end reads with a read length of 100 bp were generated (see Table 1). After quality checks, trimming of adapter, and size selection, *de novo* assembly was performed using Trinity [12] and 278,085 transcripts were reconstructed. To reduce redundancy and potential assembly errors, we clustered 278,085 transcripts into 145,738 unigenes using CD-HIT [13], and then filtered out those likely artifact transcripts with its FPKM (Fragments Per Kilobase per Million mapped fragments) values less than 1. As a result, a final high-quality dataset of 67,923 transcripts longer than 200 bp with an average length of 685 bp and an N50 of 995 bp was obtained (see Table 2). The size distribution for them is shown in Figure 1a. To evaluate the quality of the assembly, we randomly selected six transcripts to design primer pairs for RT-PCR amplifications. In this experiment, 5 out of 6 primer pairs experimentally resulted in bands of the expected sizes, and the identity of all five PCR products were confirmed by Sanger sequencing (data not shown). In addition, we analyzed the sequencing bias via detecting random distribution of reads in ORF from the assembled transcripts (see Figure 1b). Although the 3’ ends of all ORFs contained relatively fewer numbers of reads, other positions of all ORFs showed greater numbers and more even distribution. These experimental validation and data analyses suggest that the quality of our dataset is comparable to similar reports in other non-model plant species [4,14].

**Table 1:** **Summary of the sequencing data of the** ***C. taliensis*** **transcriptome**

	**Tender shoot**	**Young leaf**	**Flower bud**	**Flower**
**Original data**				
Number of reads^*^	83.26	62.51	32.00	63.69
Insert size (bp)	280	280	280	280
Total (Gb)	8.41	6.31	3.23	6.43
**After filtering** ^**^				
Number of reads^*^	77.69	57.89	29.58	58.91
Total (Gb)	6.82	5.00	2.50	5.05
**Used for analyses**				
Total (Gb)	19.37			

^*^Units: million;

^**^The reads with quality score < 20 and length < 25 bp were excluded.

**Table 2:** **Summary of sequence assembly and function annotation of the** ***C. taliensis*** **transcriptome**

	**Number of sequences**
**Assembly**	
Total number of unigenes	67,923
Total bases (Mb)	46.55
Mean unigene length (bp)	685
N50 (bp)	995
Number of unigene ( **≥**500 bp)	30,033
Number of unigene ( **≥**1 kb)	13,799
**Annotation**	
**Protein database searches**	
Transcript BLASTx against NR	38,947
Transcript BLASTx against UniRef90	39,111
Transcript BLASTx against TAIR10	33,607
Transcript BLASTx against KOG	22,350
Transcript BLASTx against Pfam	20,748
All annotated transcript	39,475
Transcripts matching all five databases	13,698
**Functional classification and pathway mapping**	
Transcripts annotated with Gene Ontology (GO) terms	24,988
Transcripts assigned with Enzyme Commission (EC) numbers	8,936
Transcript annotations against KEGG	13,134

### Functional annotation of *C. taliensis* transcriptome

**Figure 2:**
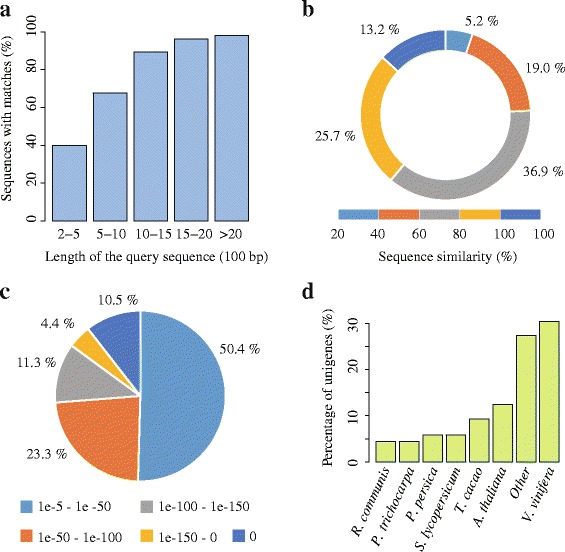
Characteristics of the homology search of unigenes against the NR database. **(a)** Effects of query sequence length on percentage of significant matches. The cut-off value was set at 1.0e-5. The proportion of sequences with matches in the NR database at NCBI is greater among the longer assembled sequences. **(b)** Similarity distribution of the best BLAST hits for each unigene. **(c)** E-value distribution of the top BLAST hits for each unigene. **(d)** Species distribution is shown as the percentage of the total homologous sequences.

To predict and analyze the function of the 67,923 transcripts, all transcripts sequences were first aligned against those sequences in the NCBI non-redundant (NR) protein database using BLASTx. A total of 38,947 significant BLAST top hits were returned with a cut-off E-value of 1e-5 (57.3% of all transcripts; see Table 2 and Additional file 1). As reported in the previous study [4], the length of transcript sequences is crucial in determining the efficiency of BLAST searches. Our results showed that 98% of the matching efficiency was observed for sequences longer than 2,000 bp, whereas the matching efficiency decreased to about 68% for those ranging from 500 to 1,000 bp and to 40% for sequences between 200 to 500 bp (see Figure 2a). The similarity distribution of the top hits in the nr database displayed that 38.9% of the mapped sequences had similarities higher than 80%, while 61.1% of the hits had similarities ranging from 20% to 80% (see Figure 2b). The E-value distribution had a comparable pattern with 49.6% of the mapped sequences with high homologies (smaller than 1e-50), whereas 50.4% of the homologous sequences ranged between 1e-5 and 1e-50 (see Figure 2c). For species distribution, 30.4% of the distinct sequences had the top matches (first hit) trained with sequences from the *Vitis vinifera*, followed by the *Arabidopsis thaliana* (12.4%), *Theobroma cacao* (9.3%), *Solanum lycopersicum* (5.9%) and *Prunus persica* (5.8%) (see Figure 2d).

To further obtain detailed descriptions and improve the annotations of the *C. taliensis* transcriptome, another three high-annotate protein databases, including UniRef90, TAIR10 and KOG, were also used to perform additional BLAST alignments with BLASTx (≤1e-5). In the case of UniRef90, a total of 39,111 matches (57.6% of all transcripts) were obtained (see Table 2). Compared against the TAIR10, containing a complete reference genome and comprehensively annotated gene sequences for the model plant *Arabidopsis thaliana*, a total of 33,607 (49.5% of all transcripts) obtained significant BLAST matches (see Table 2). Blast searches against the KOG databases revealed that 22,350 (32.9% of all transcripts) had the best hit and were assigned to 24 functional categories when E-value was less than or equal to 1e^−5^. Among the 24 KOG categories, the cluster for “general function prediction” represents the largest group (4,938, 22.1%), followed by “Posttranslational modification, protein turnover, chaperones” (2,306, 10.3%) and “Signal transduction mechanisms” (1836, 8.2%). The following categories, including “Nuclear structure” (20, 0.09%), “Extracellular structures” (100, 0.45%) and “Cell wall/membrane/envelope biogenesis” (187, 0.84%), were the smallest groups (see Additional file 2). Notably, the category of secondary metabolism made up 3.15% (704 of the functional genes) in the annotated transcripts due to the abundance of secondary metabolites in *C. taliensis*.

**Figure 3:**
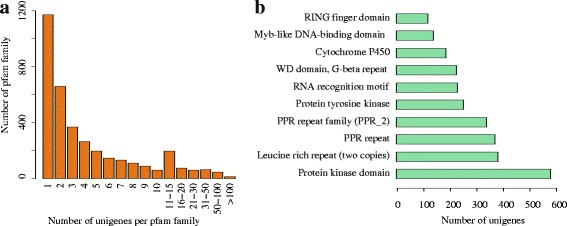
Protein families in *C. taliensis* transcriptome. **(a)** The number of Pfam domains/families versus the occurrence of *C. taliensis* transcripts contained in each domain/family. **(b)** The 10 most abundant protein families in *C. taliensis.*

**Figure 4:**
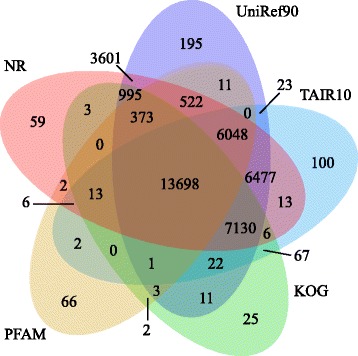
Venn diagram showing the BLAST results of *C. taliensis* transcriptome against five databases. *De novo* reconstructed transcript sequences were used to search against public databases including NR, UniRef90, TAIR10, KOG and PFAM. The number of transcripts that have significant hits against the five databases is shown in each intersection of the Venn diagram.

Considering that the information of conserved domains within a gene was indicative of deducing genes’ function, we performed the annotation of potential domains inside the assembled transcripts. To facilitate this procedure, the open reading frame (ORF) for each transcript was first extracted using a set of programs included in the Trinity package (see Methods), and then all the transcripts with predicted ORFs were searched against the PFAM database using profile hidden Markov model methods. Overall, a total of 20,748 transcripts were categorized into 3,707 domains/families. Figure 3a shows the size distribution of each domains/families, suggesting that most domains were found to contain a small number of transcripts, with a small proportion seeming more frequently. Based on the frequency of the occurrence of transcripts contained in each Pfam domain, we ranked the Pfam domains/families and listed the top ten abundant domains/families in Figure 3b, with hit results similar to the previous study [5]. Among these domains/families, “protein kinase domain” and its subclass “protein tyrosine kinase” are known to regulate the majority of cellular pathways, proteins with “leucine-rich repeats” domain are recognized to be frequently involved in the formation of protein–protein interactions, and “PPR repeat” has been reported to be a large protein family in plants with versatile functions [15]. Other protein families, such as “RNA recognition motif”, “WD domain, G-beta repeat”, and “cytochrome P450”, which have some basic functions in plants, were also found in the top ten of the list. Taken together, 39,475 transcripts got the best hits with known proteins in at least one of the five databases, and 13,698 transcripts exhibited the similarity to proteins in all of the five databases (see Figure 4 and Table 2).

### GO classification and KEGG pathway mapping

To functionally classify the *C. taliensis* transcripts, gene ontology (GO) terms and enzyme commission (EC) numbers were assigned to each transcript using Blast2GO based on the best BLASTx hit from the NR database. Of the 38,947 transcripts with NR annotation, a total of 24,988 transcripts were assigned to 137,459 GO terms. The distribution of GO terms for molecular functions, biological processes and cellular components is shown in Additional file 3. For the biological process classification, genes involved in the “cellular process” (GO: 0009987) and “metabolic process” (GO: 0008152) were highly represented. For the molecular function classification, “catalytic functions” (GO: 0003824) was the most enriched GO term, followed by “binding functions” (GO: 0005488). For the cellular component, the major categories were “cell” (GO: 0005623), “cell part” (GO: 0044464) and “organelle” (GO: 0043226). As described previously [16], the Enzyme Commission number (EC number) is a numerical classification scheme for enzymes. Of the 24,988 sequences annotated with GO terms, 8,936 sequences were assigned with EC numbers (see Table 2). To fully identify the EC numbers and biological pathways that are active in *C. taliensis*, all the assembled sequences were again assigned with Kyoto Encyclopedia of Genes and Genomes (KEGG) orthology (KO) identifiers using KEGG Automatic Annotation Server (KAAS) with the single-directional best hit information method, and subsequently mapped to pathways and enzymes using the KEGG API. As a result, we assigned 13,134 sequences to 201 KEGG pathways (see Additional file 4). The pathways with the most representation by the unique sequences were ribosome (769 members); carbon metabolism (659) and Oxidative phosphorylation (417). These annotations will undoubtedly provide valuable resources for investigating specific processes, functions and pathways during the tea development.

### Identification of genes for secondary metabolic pathways involved in tea quality and comparative analysis with *C. sinensis*

**Figure 5:**
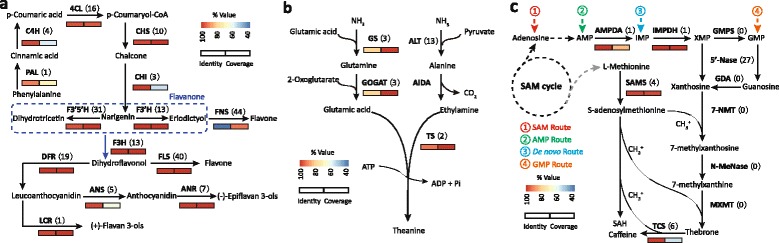
Unigenes involved in the three metabolic pathways. The number in parenthesis means the number of unigenes identified in *C. taliensis*, and the color bar represents the identity and coverage of the best unigene (Additional file 5) detected in *C. taliensis* against its corresponding gene in *C. sinensis*. **a)** Flavonoids biosynthesis pathway. **b)** Theanine biosynthesis pathway. **c)** Caffeine biosynthesis pathway.

The quality of tea, including color, smells and flavor, is a sum of all active components in tea. Therein, the most important components involved in taste and nutritional values are some secondary metabolites, such as flavonoid, amino acids, and alkaloids [17,18]. To reconstruct these vital secondary metabolic pathways in *C. taliensis* and provide valuable gene resources for the genetic improvement of cultivated tea trees, we performed the pathway analysis for the biosynthesis of three important secondary metabolites, including catechins, theanine and caffeine. As a result, most of the genes encoding the enzymes involved in these three metabolic pathways were detected in this study (see Figure 5 and Additional file 5). Relatively low homology was observed for some key genes, such as *TCS*, *FNSII* and *F4’ST*, when compared with previously cloned genes in *C. sinensis*. These differences may lead to the molecular evolutionary alterations between cultivated tea and *C. taliensis*.

#### Flavonoid biosynthesis pathway

Flavonoids are the major secondary metabolites with diverse biological activities in tea tree. They have myriad types, and play an important role in determining the quality of tea, especially the catechins, which are the most representative component of flavonoids constituting more than 70-80% of all polyphenol contents [19]. Based on the *de novo* assembly and functional annotation of the *C. taliensis* transcriptome, we identified the transcripts encoding the key enzymes involved in flavonoid pathway of *C. taliensis* (see Figure 5a). Our results showed that most of transcripts identified had multiple copies. For example, numbers of the transcripts from genes encoding Cinnamate 4-hydroxylase (*C4H*), chalcone isomerase (*CHI*), and dihydroflavonol 4-reductase (*DFR*) were 4, 3, and 19, respectively.

#### Theanine biosynthesis pathway

Theanine (γ-glutamylethylamide) is the most abundant free amino acid and a kind of unique component in tea tree [4,20]. It has several positive effects, such as making people relaxed [21,22], improving memory and attention [23] and antagonizing with caffeine [24]. Previous studies suggest that theanine is synthesized from glutamic acid and ethylamine derived from decarboxylation of alanine by theanine synthetase (TS), which is highly homologous to glutamine synthetase (GS) [4,25]. To form the glutamate and ethylamine, a number of additional enzymes are involved: GS, glutamate synthase (GOGAT), glutamate dehydrogenase (GDH), alanine transaminase (ALT), and alanine decarboxylase (AIDA). In the annotated transcriptome, most of the genes involved in this pathway were detected except for *AIDA*, which is specific in tea tree and never reported in other plants. Here we totally identified four transcripts for S-adenosylmethionine decarboxylase (*SAMDC*), which share similar domains with *AIDA*. A total of 3, 3, 3, 13 and 2 transcripts, having similarities with *GS*, *GAGOT-Fe*, *GDH*, *ALT* and *TS*, respectively, were also found in the transcriptome (Figure 5b; Additional file 5).

#### Caffeine biosynthesis pathway

Caffeine (1, 3, 7-trimethylxanthine) is another representative secondary metabolites derived from purine alkaloid in plant [26]. It was probably absorbed as a defense element during plant evolution [27]. With both protective and deleterious effects, caffeine has been prominent in research communities for decades and still aroused great interest of world-wide scientists for the industrialization of caffeine biosynthesis and a low-caffeine tea. Although caffeine biosynthesis pathway has been widely known in *C. sinensis*, however, the existing knowledge on the pathways and enzymes involved in caffeine biosynthesis in other *Camellia* species, such as *C. taliensis*, is still limited. As reported previously [28], the precursor for caffeine biosynthesis is xanthosine, which is catalyzed by three methylations and one nucleosidase reaction to form the caffeine: xanthosine → 7-methylxanthosine → 7-methylxanthine → theobromine → caffeine. The xanthosine is formed from IMP by inosine-5’-monophosphate dehydrogenase (IMPDH) [29]. Based on the transcriptome annotation, in this study, we identified most of the genes encoding enzymes involved in caffeine biosynthesis pathway. Figure 5c shows the caffeine biosynthesis pathway reconstructed based on the determined transcripts, and Additional file 5 lists all the transcripts identified above.

To assess sequence homology between *C. taliensis* and *C. sinensis*, 24 previously cloned genes that are related to the important secondary metabolic pathways in *C. sinensis* (fourteen for flavonoids biosynthesis, six for theanine biosynthesis, and four for caffeine biosynthesis), were downloaded and aligned against the corresponding transcripts identified in the *C. taliensis* transcriptome using BLASTALL package. The detailed information including gene names, alignments and GenBank Accession Numbers is given in Additional file 5. Our results showed that almost all identified transcripts were full-length (fl)-cDNA or approximately fl-cDNA, such as *F3’5’H*, *IMPDH*, and *F3H* (see Figure 5 and Additional file 5). To the best of our knowledge, this is the first time to obtain so large number of high-quality fl-cDNA sequences in the genus *Camellia*, which will greatly contribute to the *in vitro* synthesis of important secondary metabolites for tea industry. Comparisons with the genes previously cloned in *C. sinensis* revealed noteworthy variations between these two species. For example, in flavonoids biosynthesis pathway, some genes, such as *PAL* and *LCR*, were fairly conserved with high identities of more than 90%, while great sequence differences were found in *FNSII* and *F4’ST* with identities lower than 38% and 65%,respectively. Relatively high sequence similarity (~80%) was found in unigenes related to theanine biosynthesis. In caffeine biosynthesis pathway, all genes associated with the reactions before the synthesis of xanthosine were remarkably conserved, and the utmost dissimilarity was observed in unigenes encoding TCS. Although one unigene of *TCS*, CtaTrans18792, had a high identity of 99%, however, the coverage of this transcript was only 55.6%. All these variations were further confirmed by re-mapping the sequencing reads to the assembled transcripts. At the transcriptome level, the gene variations detected above are probably the reasons for the content differences of important phytochemical components between *C. taliensis* and *C. sinensis* [30-32].

### Gene expression profiling among different developmental stages

**Figure 6:**
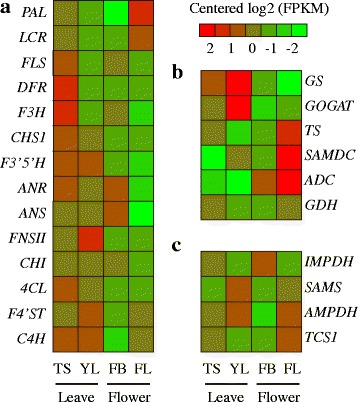
Expression pattern of candidate genes involved in different biosynthesis pathways. **a)** Flavonoids biosynthesis pathway. **b)** Theanine biosynthesis pathway. **c)** Caffeine biosynthesis pathway. (TS: tender shoots; YL: young leaves; FB: flower bud; FL: flower).

To examine the expression pattern of genes encoding enzymes involved in important secondary metabolic pathways at different developmental stages, we calculated the FPKM values for the fourteen flavonoid biosynthesis-related genes (*PAL*, *C4H*, *4CL*, *CHS*, *CHI*, *F3’H*, *F3’5’H*, *FNSII*, *FLS*, *DFR*, *LCR*, *ANS*, *ANR* and *F4’ST*), the six theanine biosynthesis involved genes (*TS*, *GS*, *GDH*, *ADC*, *SAMD*C and *GOGAT-Fe*) and the four caffeine biosynthesis-related genes (*IMPDH*, *SAMS*, *AMPDA* and *TCS*) (see Figure 6). We observed that most of these genes associated with flavonoids biosynthesis pathway exhibited a similar expression pattern with higher levels in tender shoots but slightly decreased in young leaves (see Figure 6a), indicating that the flavonoid biosynthesis actively occurs in tender shoots. These observations are consistent with previous results obtained in *C. sinensis* [33,34]. It is worth noting that some genes, such as *PAL*, *ANS*, *ANR* and *LCR*, exhibit relatively higher expression levels in either flowers or flower buds, reinforcing previous studies suggesting that flavonoid biosynthesis may be involved in flower coloration [34,35].

Genes involved in theanine biosynthesis can be mainly divided into two types: glutamic acid synthesis related (*GS*, *GOGAT-Fe*, and *GDH*) and ethylamine synthesis related (*ALT* and *AIDA/SAMDC/ADC*). Expression analysis revealed that genes related to glutamic acid synthesis were highly expressed in young leaves except for *GDH* (see Figure 6b). By contrast, genes involved in ethylamine synthesis, including *ADC* and *SAMDC*, showed the highest level of expression in flowers (see Figure 6b). A similar pattern was also observed in *TS*. Previous studies confirmed that theanine was primarily synthesized in the root of tea trees, and then transformed into the tender shoots through the xylem [4]. Combined with the former discovery that tea flower has a comparable concentration of theaine [18], it is likely that a larger portion of theanine in tea flower may be synthesized *in situ* compared with other tissues.

Our analyses of expression levels of genes encoding *SAMS*, *AMPDA*, *IMPDH* and *TCS* in caffeine biosynthesis showed that all these studied genes were expressed in a constitutive manner and detected with a relatively high expression level in vegetative organ (see Figure 6c). This observation suggests that caffeine biosynthesis vigorously act in tender shoots and young leaves, consistent to an earlier study [33]. It is worth mentioning that another expression peak of all genes was found in flower tissues, especially for *TCS*, of which the expression level is much higher than that in other tissues. Together with the former discovery in *C. sinensis* [34], our results further demonstrate that the flower is probably another important place for the caffeine biosynthesis.

### Validation of unigenes and gene expression profiling using RT-qPCR

**Figure 7:**
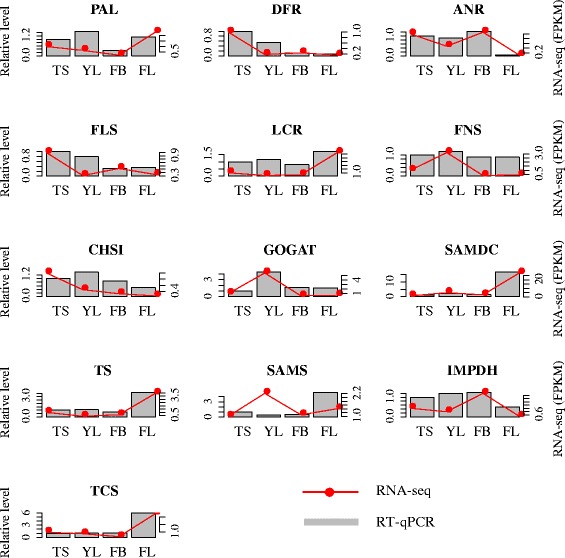
Quantitative RT-qPCR validations. A total of 13 genes were selected for the quantitative RT-qPCR experiments. Of them, *PAL*, *DFR*, *ANR*, *FLS*, *LCR*, *FNS* and *CHSI* were for flavonoids biosynthesis pathway, *GOGAT*, *SAMDC* and *TS* belong to theanine biosynthesis pathway, and *SAMS*, *IMPDH* and *TCS* were from caffeine biosynthesis pathway.

In order to experimentally validate the reliability of unigenes obtained from the assembled transcriptome and profiling of gene expression obtained by RNA-Seq data, a total of 13 key unigenes involved in the biosynthesis of flavonoids, theanine and caffeine were selected for RT-qPCRs (Figure 7). The detailed results of the selected unigenes, IDs and primer pairs used in this study are shown in Additional file 6.

RT-PCR amplification was performed to confirm the suitability of the used primer pairs, showing that all 13 selected unigenes were successfully amplified with single bands with the expected sizes (Additional file 6). The results suggest that the assembled transcripts are reliable and the designed primer pairs are suitable for the subsequent expression experiments. Based on the delta-delta Ct (2^-ΔΔCt^) method, relative expression levels of the selected unigenes were calculated and compared among the four different tissues. All the expression patterns of these genes detected by RT-qPCR were mainly consistent with those from RNA-Seq data except for *SAMS*. This gene exhibited the highest expression level in the flower detected by RT-qPCR, while the highest expression level was observed in young leaves with the RNA-Seq data (Figure 7). Overall, RT-qPCR experiments confirmed that the unigenes obtained from the assembled transcriptome are trustworthy and gene expression profiles from RNA-Seq data should be believable.

### Comparative analysis of stress-resistance related genes in *C. taliensis* and *C. sinensis*

Similar to other economic crops, tea yields are often affected by biotic and abiotic stresses, such as the pathogens *Exobasidium vexans* Masse, *Phyllosticta gemmiphliae* Chen et Hu, and *Gloeosporium theae-sinensis* Miyake, or harsh environmental conditions. To adapt the rapidly changing environments, plants have developed a variety of defense mechanisms during the process of evolution, including signaling molecules [36], genes encoding transcription factors [37] and relative gene family expansion [38]. To date, *C. taliensis* has become seriously endangered due to an overexploitation driven by economic incentives. To better conserve and make good use of germplasm resources of this wild tea species, we identified and characterized the putative stress resistance-related genes in both the published transcriptome of *C. sinensis* and sequences of *C. taliensis* assembled in this study. Our goal is to have a first glimpse at the variation of resistance genes among these two species using RNA-Seq data and provide some references and gene resources for the tea tree study in the future.

#### Identification of transcription factors in C. taliensis

**Figure 8:**
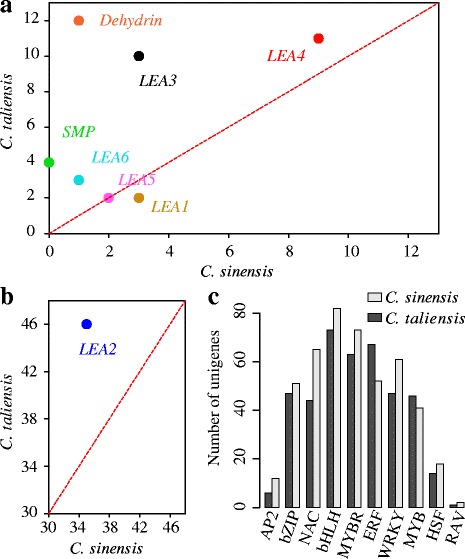
Comparative analysis of stress resistance related genes between *C. taliensis* and *C. sinensis* transcriptome*.* **a)** The members of *LEA* family identified in *C. taliensis* and *C. sinensis*. The y-axes represent the numbers of *LEA* members found in *C. taliensis*, while x-axes shows the number identified in *C. sinensis*. Red dashed line means the number of *LEA* members that are equivalent in these two species. **b)** The largest *LEA* family identified in *C. taliensis* and *C. sinensis*. **c)** The number of cold tolerance related TFs identified from *C. taliensis* and *C. sinensis*.

Transcription factors (TFs) are kinds of DNA-binding proteins that can activate or repress gene expression through specific interactions with *cis*-acting elements in promoters of eukaryotic genes [39]. The activation of a large number of stress-related genes is mediated by specific TFs [40]. Over the past decades, many TFs related to stress responses have been identified from different plants [41], but little has been known in tea tree so far. To identify putative genes that could be involved in freezing tolerance, we searched for members of the TFs families potentially involved in cold resistance, including *AP2*, *bZIP*, *NAC*, *BHLH*, *WYBR*, *ERF*, *WRKY*, *MYB*, *HSF* and *RAV*, in both *C. taliensis* and *C. sinensis* using a domain-based pipeline (see Methods). As a result, a total of 408 and 457 members were detected in *C. taliensis* and *C. sinensis*, respectively (see Additional file 7a). Of them, *RAV* represents the smallest group with only one and two members in *C. taliensis* and *C. sinensis*, respectively. While *bHLH* is the most preponderance class with 73 and 82 members were identified in *C. taliensis* and *C. sinensis*. Comparative analysis showed that some TF family sizes of *C. sinensis* are slightly larger than those in *C. taliensis*, but others are in opposite (see Figure 8c). For example, *AP2*, *NAC* and *WRKY* respectively have 12, 65 and 61 copies in *C. sinensis*, while 6, 44, 47 members in *C. taliensis*. Interestingly, the number of *ERF* gene family in *C. taliensis* is 67, which is much larger than those in *C. sinensis* (52 copies).

#### LEA gene family

Late embryogenesis abundant protein (LEA) was firstly identified in cotton [42]. After that, it was successively discovered in a variety of plants [43,44]. LEA proteins have been found to be highly hydrophilic with a high ratio of glycine or other small amino acids [45], which functions extreme desiccation tolerance and stress resistance in plants, especially for cold tolerance. Previous studies have demonstrated the important roles of LEA proteins in protecting plants from abiotic stress, for example, the over-expression of wheat *LEA* (*WCOR410*) increased the freezing tolerance in strawberry leaves [46]. To further identify putative genes that could be involved in cold tolerance in *C. taliensis* and *C. sinensis*, we detected the LEA members in these two species based on the searches of the conserved LEA domains. Comparative analysis showed that the total number of LEA genes in *C. taliensis* was much larger than that in *C. sinensis* (nearly 1.7-fold; see Figure 8a; b and Additional file 7b). Except for subfamily *LEA1*, *LEA4* and *LEA5*, the size of which are approximately equal between these two species, the other classes of LEA family are much abundant in *C. taliensis*. Notably, we found that *LEA2* was the most abundant class in tea tree with 46 and 35 members in *C. taliensis* and *C. sinensis*, respectively. This copy number variation of *LEA* members between these two species may lead to the high altitude and low temperature niche adaptation in *C. taliensis*.

#### NBS-LRR related transcripts

The majority of plant disease resistance genes are sequences encoding proteins with a nucleotide-binding site and C-terminal leucine-rich repeat (NBS-LRR proteins) [47,48]. They have been shown to be involved in plant resistance to biotic stresses, such as fungus, bacteria, viruses and nematodes [49], and have been cloned in several plants. Traditionally, based on the presence or absence of a Toll/Interleukin-1 Receptor (TIR) domain at the N-terminus, this class can be divided into two subgroups, TIR-NBS and non-TIR-NBS proteins [50]. A coiled-coil (CC) motif may also exist in the N-terminal region of most of the members without TIR domain [47,51]. In this study, based on the HMMsearch, 102 and 148 genes encoding NBS domains were detected in *C. taliensis* and *C. sinensis*, respectively (see Table 3 and Additional file 7c). To classify these NBS-encoding genes, all the members were evaluated for CC or TIR domains in N terminus as well as LRRs in C terminus. Result showed that 52 and 126 genes had only NBS domain, representing the most abundant class in tea tree, and accounted for 51% and 85% in *C. taliensis* and *C. sinensis*, respectively. Except for the solo-NBS domain containing transcripts, four and one genes were predicted to encode N-terminal TIR domains in *C. taliensis* and *C. sinensis*, respectively. Wherein, one of these genes in *C. taliensis* has a complete LRR domain. 23 of 102 genes in *C. taliensis* and 13 of 148 genes in *C. sinensis* were identified with CC motif, of which 14 and 11 were detected without the LRR domain, respectively. Additionally, 23 and 8 genes only had NBS-LRR domains in *C. taliensis* and *C. sinensis*. Overall, apart from the class with only NBS domain, the amount of other types of *R* genes is much fewer in cultivated tea tree*.* This probably derived from the short length of transcripts in *C. sinensis* [4].

**Table 3:** **Summary of the disease resistance genes between** ***C. taliensis*** **and** ***C. sinensis*** **transcriptome**

	***C. taliensis***	***C. sinensis***
CC-NBS	14	11
CC-NBS-LRR	9	2
TIR-NBS	3	1
TIR-NBS-LRR	1	0
NBS-LRR	23	8
NBS	52	126
Total	102	148

## Conclusions

This study presents the first transcriptome of *C. taliensis,* a closely related wild species of *C. sinensis*. A total of ~24 Gb reads was generated and assembled into 67,923 unigenes, of which 39,475 could be functionally annotated. The analysis of related pathways identified the majority of candidate genes involved in important secondary metabolic pathways responsible for tea quality in this species. Comparisons with corresponding genes cloned in *C. sinensis* revealed that most of them were relatively conserved except *FNSII*, *F4’ST* and *TCS*. Moreover, the obtained information on the transcription factors and NBS-related transcripts in *C. taliensis* will facilitate the discovery of other stress resistance genes. Overall, the large EST sequences reported here will serve as a valuable resource to accelerate the genetic improvement of tea trees and the industrialization of tea-related products.

## Methods

### RNA preparation

Four different tissues including tender shoots, young leaves, flower buds and flowers of *C. taliensis* were harvested from Tea Research Institute, Yunnan Academy of Agricultural Sciences (TRI, YAAS), snap-frozen immediately and then stored at −70°C until processing. These materials were divided into two parts, one for sequencing and another for RT-qPCR experiment with three replicates. Total RNA was extracted by modified CTAB method [52] and treated with DNase I. After further extraction to remove the protein of DNase I, RNA integrity was confirmed using the Agilent 2100 Bioanalyzer with a minimum integrity number value of 8.

### cDNA library construction and sequencing

The poly (A)^+^ RNA (mRNA) was isolated from 20 μg of the total RNA pool using Dynal oligo(dT) 25 magnetic beads according to the manufacturer’s protocol. Followed by purification, the mRNA was fragmented into smaller pieces by the fragmentation buffer (Ambion). Then the cleaved RNA fragments were used for first-strand cDNA synthesis using SuperScript III reverse transcriptase and N6 random hexamers. Subsequently, second strand cDNA was synthesized using RNase H and DNA polymerase. These cDNA fragments were further processed by an end repair and the ligation of adapters according to the manufacturer’s protocol. The products were purified and enriched with PCR for preparing the final sequencing library. The cDNA library was detected by Agilent 2100 Bioanalyzer. The cDNA library was sequenced from both 5′ and 3′ ends using the Illumina HiSeq 2000 platform by following the manufacturer’s instructions. The fluorescent image processing, base-calling and quality value calculation were performed by the Illumina data processing pipeline 1.4, in which 100 bp paired-end reads were obtained.

### Data-preprocessing and *de novo* assembly

The raw reads were first pre-screened to remove adaptors, poly-A tails and contaminants by Seqclean. Low-quality (phred score < 20) and short (length < 25 bp) reads were trimmed by SolexaQA package (−h 20; -l 25). The trimmed and size-selected reads were then *de novo* assembled using Trinity package.

### Redundancy removal and assembly assessment

To reduce the unavoidable redundancy produced by Trinity assembly, clustering analysis with CD-HIT software was performed. To ensure the quality and avoid potential assembly errors, the transcripts with FPKM values less than 1 were also removed. The remaining dataset of transcripts was then used to count the basic assembly statistics and perform the downstream analysis. In order to evaluate the quality of assembly, primers were designed for six singletons selected randomly. Then RT-PCR amplification was performed and the PCR products were sequenced by Sanger sequencer. The sequencing bias via detecting random distribution of reads in ORF of the assembled transcripts was also analyzed to assess the reliability of the assembly.

### Data deposit

The data sets of sequencing reads and the assembled sequences have been deposited in the National Center for Biotechnology Information database (NCBI: www.ncbi.nlm.nih.gov), and can be retrieved under the BioProject accession number PRJNA274899.

### Gene annotation and classification

All assembled non-redundant unigenes (≥200 bp) were annotated by aligning against the NR, UniRef90, TAIR10, and KOG databases by BLASTALL package with the significant threshold of E-value ≤ 1e-5. Conserved domain-based annotation was also performed to identify potential conserved domains/families. To facilitate this procedure, the likely open reading frame (ORF) for each transcript was first extracted using *TransDecoder* program, which is included in the Trinity software distribution. Then the predicted amino sequences were searched against the Pfam database using HMMER3 software. To annotate transcripts with GO terms, the best Blastx hit from NR database for each transcript was submitted to BLAST2GO platform, and GO terms for each transcript were retrieved based on the relationship between gene names and GO terms. EC number was assigned and parsed based on the BLAST2GO results. To determine metabolic pathways, Kyoto Encyclopedia of Genes and Genomes (KEGG) mapping was used. The sequences with corresponding ECs obtained from Blast2GO were mapped to the KEGG metabolic pathway database. To further improve the pathway annotation and identify the BRITE functional hierarchies, assembled sequences were also submitted to the online KEGG Automatic Annotation Server (KAAS; http://www.genome.jp/kegg/kaas/) with bi-directional best hit (BBH) method. The output includes KO assignments and KEGG pathways that are involved with the KO assignments.

### Gene expression analysis

The expression level of each unigene was measured with a FPKM method. Briefly, reads were first aligned against each transcript of *C. taliensis* using TopHat v2.0.10, and the FPKM (fragments per kilobase of exon per million fragments mapped) expression value was then calculated using Cufflinks v2.1.1 with default parameters.

### Experimental validation of candidate genes and levels of gene expression by using RT-qPCR

Thirteen important unigenes potentially involved in the three major biosynthesis pathways of flavonoids, theanine and caffeine were selected for qRT-PCR experiments. Gene-specific primer pairs were designed using Primer primer 5.0 software (Premier Biosoft International), and total RNA was isolated from tender shoots, young leaves, flower bud and flower from *C. taliensis* using a modified CTAB method, respectively. After treated with DNase I (Tiangen, China), one microgram of RNA was used in reverse transcription with the SuperScript VILO cDNA Synthesis Kit (Invitrogen) according to the manufacturer’s guidelines. The standard curve for each gene was conducted in several dilutions of cDNA, then real-time qPCR was performed using Multicolor Real-Time PCR Detection System (Bio-Rad) with conditions for all reactions were 95°C for 30s, 40 cycles of 95°C for 15 s, followed by 60°C for 30 s, and 72°C for 20 s. Melting curve and agarose gel electrophoresis analysis were performed to confirm the PCR specificity. The translation elongation factor 1-alpha (TEF) gene was selected as an internal standard for normalization, and three biological replicates were completed for each gene. The relative expression levels for each unigene were in the different tissues calculated by using the delta-delta Ct (2^-ΔΔCt^) method. All data were expressed as the mean ± SD after normalization.

### Transcription factor and LEA gene family analysis

To facilitate the transcription factor prediction, a high-quality and non-redundant protein dataset was first constructed based on the following steps: 1) predicting the coding sequence (CDS) and corresponding peptide sequence by ESTscan with CDS length > = 150 and score > = 200; 2) filtering out those proteins whose 'x' content is greater than 5%; and 3) clustering proteins using blastclust with identity > = 0.95 and coverage > = 0.9. The resulted protein dataset was subsequently searched against the domain of ten plant transcription factors, including AP2 (PF00847), BZIP (PF00170), NAC (PF02365), bHLH (PF00010), MYB (PF00249), Auxin_resp (PF06507), SWIRM (PF04433), WRKY (PF03106), HSF (PF00447) and B3 (PF02362), using hmmsearch program supplied in the HMMER package (version 3.0) with default parameters. To further validate the occurrence of these TF-related domains, PlantTFDB transcription factor prediction server (version 3.0; http://planttfdb.cbi.pku.edu.cn/) was also used. For the identification of LEA gene family, a similar strategy was adopted. Briefly, eight Hidden Markov Model (HMM) profiles for LEA_1 (PF03760), LEA_2 (PF03168), LEA_3 (PF03242), LEA_4 (PF02987), LEA_5 (PF00477), LEA_6 (PF10714), Dehydrin (PF00257) and SMP (PF04927) were downloaded from the PFAM database (http://pfam.sanger.ac.uk/) and used to scan the above resulted non-redundant protein dataset using hmmsearch program. The obtained LEA transcripts were further classified into the eight categories according to the gene family information.

### Identification of the NBS-related transcripts

A complete set of NBS-related transcripts were identified with a progressive process. First, the above predicted peptide sequences for each transcript was screened against the Hidden Markov Model profile corresponding to the Pfam NBS (NB-ARC) family (PF00931; http://pfam.sanger.ac.uk/) using HMMER package. Due to the fact that the NBS-encoding resistance genes are often associated with other domains such as TIR and CC in the N-terminal region or a variable number of LRR on the C-terminal region. We detected the TIR and LRR domains that contained within the above obtained NBS-related candidate genes using HMM search again, and the TIR and LRR profiles were downloaded from the PFAM database. Both TIR and LRR domains were validated using NCBI conserved domains and Multiple Expectation Maximization for Motif Elicitation (MEME). As PFAM analysis could not identify the CC motif in the N-terminal region [47,53], we predicted the CC domain using the NCOILS program with default parameters [54].

## Additional files

Additional file 1:
**Top BLAST hits from the NCBI NR database.** BLAST results against the NCBI NR database for all the transcripts with E-value ≤ 10–5 are shown. Additional file 2:
**KOG functional classification of** ***C. taliensis*** **transcripts.** A total of 22,350 unigenes showing significant homology to the KOGs database at NCBI (E-value ≤ 1.0e-5) have a KOG classification among the 24 categories. Additional file 3:
**Gene Ontology classification of** ***C. taliensis*** **.** Gene ontology (GO) terms were assigned to *C. taliensis* unigenes based on the top hit against the NR database. The left y-axis indicates the percentage of a specific category of genes in that main category. The right y-axis indicates the number of genes in the same category. Additional file 4:
**Pathways identified in** ***C. taliensis*** **transcriptome.** 201 KEGG pathways identified in *C. taliensis* and the corresponding unigenes numbers of each pathway were showed. Additional file 5:
**List of the putative unigenes related to three secondary metabolic pathways in the** ***C. taliensis*** **transcriptome.** *C. taliensis* unigenes involved in three secondary metabolic pathways, namely flavonoids biosynthesis, theanine biosynthesis, and caffeine biosynthesis, and the alignment results between *C. taliensis* and *C. sinensis* were listed. Additional file 6:
**List of the unigenes, IDs and primer pairs used for quantitative RT-qPCR experiments.** Specific primer pairs of thirteen candidate unigenes with potential roles in three secondary metabolic pathways designed for real time RT-qPCR using the Primer premier software (version 5.0) were shown. The translation elongation factor 1-alpha (TEF) gene was used as the reference gene for internal control for normalization. Additional file 7:
**List of stress resistant related genes detected in** ***C. taliensis*** **and** ***C. sinensis*** **transcriptome.** a) Numbers of transcripts belonging to different transcription factor families in *C. taliensis* and *C. sinensis*; b) The members of LEA family identified in *C. taliensis* and *C. sinensis*; c) The list of NBS gene family detected in *C. taliensis* and *C. sinensis*.
