# Supplementary figures and images for: De novo transcriptome assembly of the wild relative of tea tree (Camellia taliensis) and comparative analysis with tea transcriptome identified putative genes associated with tea quality and stress response
Source: BMC Genomics. 2015 Apr 15;16(1):298. doi: 10.1186/s12864-015-1494-4 (PMC4404113; doi:10.1186/s12864-015-1494-4)

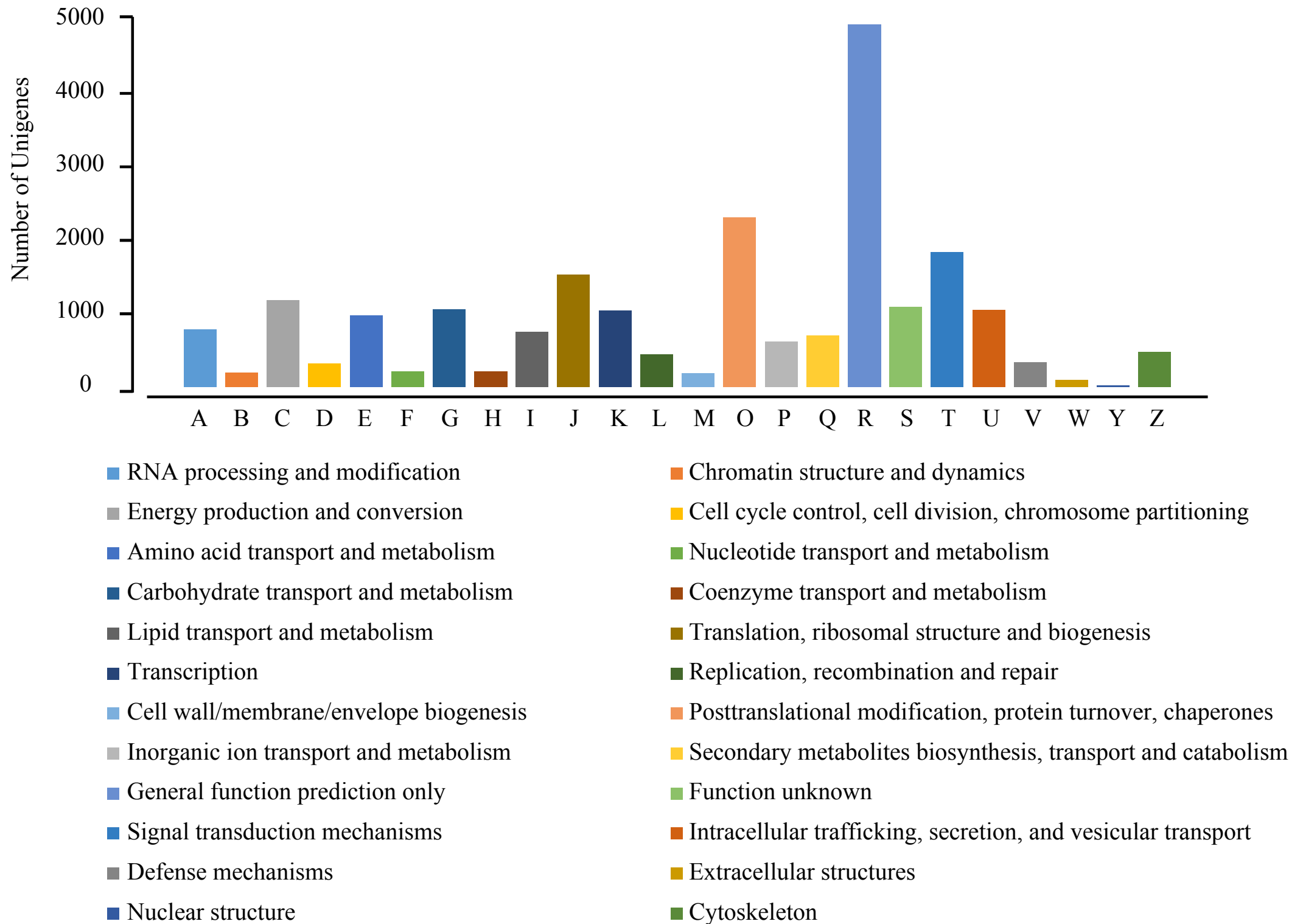

Supplement: Additional file 2: — KOG functional classification of C. taliensis transcripts. A total of 22,350 unigenes showing significant homology to the KOGs database at NCBI (E-value ≤ 1.0e-5) have a KOG classification among the 24 categories. [file 12864_2015_1494_MOESM2_ESM.pdf]

The GO classification of *C. taliensis* transcriptome

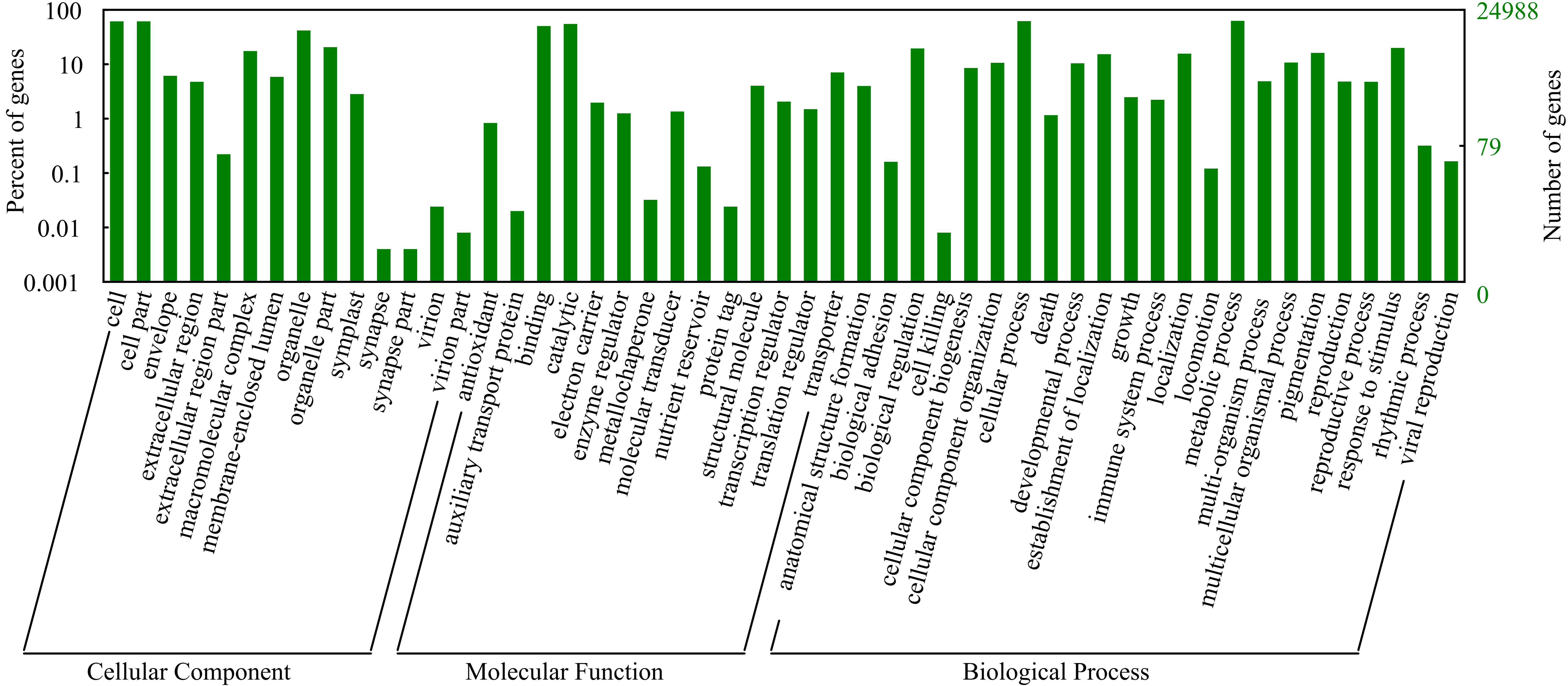

Supplement: Additional file 3: — Gene Ontology classification of C. taliensis . Gene ontology (GO) terms were assigned to C. taliensis unigenes based on the top hit against the NR database. The left y-axis indicates the percentage of a specific category of genes in that main category. The right y-axis indicates the number of genes in the same category. [file 12864_2015_1494_MOESM3_ESM.pdf]
